# Reporting of factorial trials of complex interventions in community settings: a systematic review

**DOI:** 10.1186/1745-6215-12-179

**Published:** 2011-07-19

**Authors:** Alan A Montgomery, Margaret P Astin, Tim J Peters

**Affiliations:** 1 Bristol Randomised Trials Collaboration, School of Social and Community Medicine, University of Bristol, 39 Whatley Road, Bristol BS8 2PS, UK; 2 Academic Unit of Primary Health Care, School of Social and Community Medicine, University of Bristol, 39 Whatley Road, Bristol BS8 2PS, UK; 3 School of Clinical Sciences, University of Bristol, Second Floor, Learning and Research, Southmead Hospital, Bristol BS10 5NB, UK

## Abstract

**Background:**

Standards for the reporting of factorial randomised trials remain to be established. We aimed to review the quality of reporting of methodological aspects of published factorial trials of complex interventions in community settings.

**Methods:**

We searched MEDLINE, EMBASE, PsychInfo and the Cochrane Controlled Trials Register to identify factorial randomised trials of complex interventions in community settings from January 2000 to August 2009. We also conducted a citation search of two review papers published in 2003. Data were extracted by two reviewers on 22 items relating to study design, analysis and presentation.

**Results:**

We identified 5941 unique titles, from which 116 full papers were obtained and 76 were included in the review. The included trials reflected a broad range of target conditions and types of intervention. The median sample size was 400 (interquartile range 191-1001). Most (88%) trials employed a 2 × 2 factorial design. Few trials (21%) explicitly stated the rationale for using a factorial design. Reporting of aspects of design, analysis or presentation specific to factorial trials was variable, but there was no evidence that reporting of these aspects was different for trials published before or after 2003. However, for CONSORT items that apply generally to the reporting of all trials, there was some evidence that later studies were more likely to report employing an intention-to-treat (ITT) approach (78% vs 52%), present appropriate between-group estimates of effect (88% vs 63%), and present standard errors or 95% confidence intervals for such estimates (78% vs 56%). Interactions between interventions and some measure of the precision associated with such effects were reported in only 14 (18%) trials.

**Conclusions:**

Reports of factorial trials of complex interventions in community settings vary in the amount of information they provide regarding important methodological aspects of design and analysis. This variability supports the extension of CONSORT guidelines to include the specific reporting of factorial trials.

## Background

Factorial randomised trials offer the potential of efficient evaluation of more than one intervention in a single study. The design also permits investigation of interactions between interventions - that is, whether the combined effects are less than (antagonistic) or greater than (synergistic) the additive effects one would observe if the treatments acted independently of one another. The design, analysis and reporting of factorial trials has been described previously [1,2]. Briefly, the simplest factorial design of two interventions involves allocating patients to receive both, one or other, or no intervention. Analysis "at the margins" assumes that the two treatments act independently, and uses all trial participants in estimating the effects of each treatment. However "inside the table" analysis must be used to estimate the effect of each treatment if an interaction is suspected or observed. As these analyses each involve only half of the trial participants, it is easy to see why the design is most efficient for evaluating interventions where no substantive interaction is anticipated.

Complex interventions are widely used in health services and areas of social policy, often in community settings. Evidence of effectiveness may be sought by rigorous evaulation in randomised studies. The UK Medical Research Council (MRC) published revised guidance on developing and evaluating complex interventions in 2008 [3]. It may be helpful to consider interventions as part of a complexity continuum rather than as a clear dichotomy between simple and complex. The features that make an intervention complex can include the number of interacting components, the behaviours required by those delivering or receiving the intervention, the groups or organisational levels targeted, the number and variability of expected outcomes, and any permitted tailoring of the intervention. Randomised evaluations of complex interventions often involve large and lengthy studies that require significant investment of time from investigators and participants, and money from research funders. Given this investment, a study design that enables simultaneous evaluation of multiple interventions, or components of a complex intervention, has an obvious attraction.

If we accept the notion of a continuum rather than a dichotomy between simple and complex interventions, it could be argued that most RCTs of (non-pharmacological) health care interventions involve at least some of the complexities listed above. In RCTs carried out in community settings where the key perspective is a pragmatic evaluation of effectiveness and cost-effectiveness of interventions in as close to 'usual' circumstances as possible, complex interventions are the norm. Since community settings is where the majority of health care is delivered internationally, and that such RCTs have arguably the widest generalisability, it seemed timely to focus a review on these trials. The aim of this study was therefore to conduct a systematic review of the reporting of key methodological issues in published factorial trials of complex interventions in community settings.

## Methods

### Data sources and search methods

We conducted searches of electronic databases in MEDLINE, Embase and PsychINFO and the Cochrane Controlled Trials Register from January 2000 to August 2009. The search strategy was composed of two sets that were combined with the AND operator. The first set comprised Medical Subject Heading (MeSH) and free text terms with synonyms and spelling variations to identify randomised studies. Since there is no MeSH term for factorial trials, the second set comprised the free text terms "factorial", "2 by 2" and "2 × 2". Although our particular focus was trials of complex interventions in community-based settings, reliable identification of such trials using either MeSH or free text terms was not possible, and we were unable to include sets for these features in the search strategy. The search strategy excluded animal studies and was restricted to articles published in English. We conducted citation searches of two papers describing the design, analysis and reporting of factorial trials both published in 2003 [1,2]. The full search strategy is given in Additional File 1.

### Inclusion criteria

Studies of randomised controlled trials with factorial designs were eligible. We defined factorial trials as those in which more than one intervention is evaluated, and where participants may be allocated to receive one, more than one, or no intervention. All population groups including adults, children and the elderly were of interest, as were studies in which the participants were health care professionals. The studies were restricted to those where recruitment and treatment of participants occurred in communities and primary care settings, and only those where at least one of the interventions could be considered complex as described by the UK Medical Research Council guidance [3] were included.

Studies conducted in hospital or other secondary care environments, and studies of interventions not considered complex were excluded. Regarding the latter, the MRC guidance states that there is no sharp boundary between simple and complex interventions. While exclusion of some trials was clear, such as those involving only medicinal products, others required further discussion between the authors. Table 1 gives some examples of interventions that were included/excluded. Other exclusions were pilot studies, trial protocols, non-randomised studies, secondary analyses of previously reported trials, and studies with non-clinical primary outcomes.

**Table 1:** Examples of interventions and settings included/excluded in the review

Included	Excluded
**Complex intervention**	**Simple intervention**

Dietary counselling to increase fruit and vegetable consumption	Dietary supplement tablets

Physiotherapy and exercise programme	Specific physiotherapy technique eg manipulation, ultrasound

Unsupervised physical exercise programme and telephone support	Closely supervised strength training programme

Drug + complex intervention	No complex intervention

**Community setting**	**Other setting**
Primary care/general practice/family doctor/other community setting (eg home, community centre, gym)	Hospitals/Specialist medical or research centres/University clinics

### Study selection and data extraction

One researcher (AAM) conducted the searches. Titles and abstracts were screened independently by two researchers (AAM and CE) for retrieval of full papers. Any disagreements were resolved by consensus. We developed a template for data extraction based on previous reviews of factorial [1] and crossover [4] trials. We piloted the form using a random selection of five papers, before making final modifications prior to use. We extracted data on the reporting of important methodological details. One reviewer extracted data (MPA) which were then checked by a second reviewer (AAM), and any disagreements were resolved by consensus. Authors were contacted for further information if required.

### Data analysis

Trials were categorised according to year of publication (2000-03 versus 2004-09). These dates were selected to investigate whether reporting of factorial trials improved following the publication in 2003 of two papers describing the design, analysis and presentation of factorial trials [1,2]. The proportion of trials reporting each item was determined using simple tabulations, with differences between 'earlier' and 'later' trials investigated using chi-squared statistics and differences in proportions with 95% confidence intervals. All data were analysed using Stata 11 [5].

## Results

### Study selection

**Figure 1:**
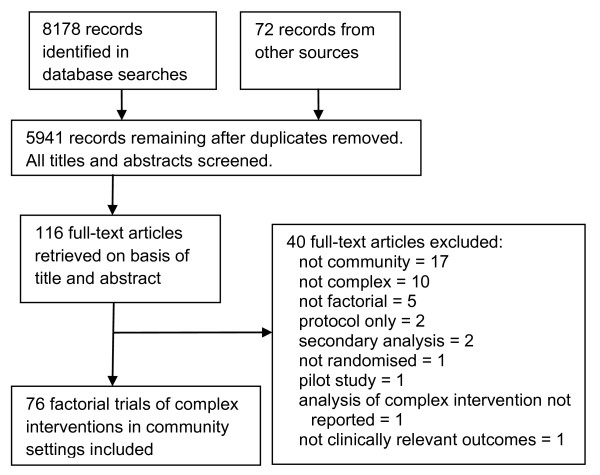
**Diagram of information flow**.

A total of 5941 titles were obtained from the search strategy, from which 116 full papers were obtained for detailed evaluation because they appeared to describe factorial trials of complex interventions in community settings (Figure 1). Of these, 40 were excluded, with the most common reasons being either that the trial was conducted in a setting other than the community, or that none of the interventions evaluated were regarded as complex. Of the 76 trials included, 49 (64%) were published after 2003.

### Characteristics of included trials

The studies we included encompassed a wide range of clinical conditions, reflecting the breadth of research based in general practice and other community settings (Additional File 2). Most of the studies involved adult participants (64/76, 84%). The median number of participants was 400 (IQR 191-1001). The term "factorial" was used in the title or abstract of 70/76 (92%) trials (Table 2). While efficient evaluation of more than one intervention is commonly the main reason for using a factorial design, followed by the ability to investigate interactions between interventions, only 16/76 (21%) of trials explicitly stated the rationale for using this design.

**Table 2:** Reporting of methodological aspect of included studies

			Year of Publication	2000-2003		2004-2009		Total	
			**Number of Trials**	**27**		**49**		**76**	

**Heading**	**Subheading**	**Methodological Descriptor**		**Frequency**	**Percent**	**Frequency**	**Percent**	**Frequency**	**Percent**

Title and abstract		Phrase "factorial" used	No	2	(7)	4	(8)	6	(8)
			Yes	25	(93)	45	(92)	70	(92)

Introduction	Background	Rationale for factorial trial established	Efficiency	3	(11)	4	(8)	7	(9)
			Interaction	0	(0)	2	(4)	2	(3)
			Both	4	(15)	3	(6)	7	(9)
			Not stated	20	(74)	40	(82)	60	(79)

Methods	Design	Number of treatments	2 × 2	24	(89)	43	(88)	67	(88)
			Other	3	(11)	6	(12)	9	(12)
	
	Allocation	Units of allocation	Individual only	20	(74)	28	(57)	48	(63)
			Cluster only	5	(19)	16	(33)	21	(28)
			Mixed	2	(7)	5	(10)	7	(9)
		
		Adequate description of randomisation (generation of sequence, execution)	No	12	(44)	24	(49)	36	(47)
			Yes	15	(56)	25	(51)	40	(53)
		
		Adequate allocation concealment	No	1	(4)	2	(4)	3	(4)
			Yes	10	(37)	21	(43)	31	(41)
			Unclear	16	(59)	26	(53)	42	(55)
	
	Sample size	Sample size calculation given	No	10	(37)	13	(27)	23	(30)
			Yes	17	(63)	36	(73)	53	(70)
		
		If yes, estimates based on analysis:	At the margins	11	(65)	27	(75)	38	(71)
			Interaction/Inside the table	1	(6)	4	(11)	5	(10)
			Unclear/Inadequate	5	(29)	5	(14)	10	(19)
	
	Statistical methods	Analytical approach	ITT	14	(52)	38	(78)	52	(68)
			Not explicitly stated	13	(48)	11	(22)	24	(32)
		
		Primary analysis correctly adjusted for design variables/baseline	No	2	(7)	2	(4)	4	(5)
			Yes	22	(81)	39	(80)	61	(80)
			Unclear	3	(11)	8	(16)	11	(15)
		
		For cluster allocations, appropriate analysis used	No	2	(29)	3	(14)	5	(18)

			Yes	5	(71)	17	(81)	22	(79)
			Unclear	0	(0)	1	(5)	1	(4)
Results	Participant flow	Reported numbers randomly assigned and followed up	CONSORT diagram	15	(56)	33	(67)	48	(63)
			No diagram but adequate text	3	(11)	2	(4)	5	(7)
			Inadequate	9	(33)	14	(29)	23	(31)
	
	Baseline characteristics	Baseline data reported in table for treatment groups	No	5	(19)	7	(14)	12	(16)
			Yes	22	(81)	42	(86)	64	(84)
		
		If yes, data reported:	At margins	3	(14)	10	(24)	13	(20)
			Inside the table	17	(77)	29	(69)	46	(72)
			Both	2	(9)	3	(7)	5	(8)
	
	Primary analyses	Descriptive data provided at follow up (eg mean & SD, N & %)	No	2	(7)	7	(14)	9	(12)
			Yes	25	(93)	42	(86)	67	(88)
		
		If yes, data reported:	At margins	8	(32)	13	(31)	21	(31)
			Inside the table	8	(32)	18	(43)	26	(39)
			Both	9	(36)	11	(26)	20	(30)
		
		Appropriate between-group estimate(s)	No	10	(37)	6	(12)	16	(21)
			Yes	17	(63)	43	(88)	60	(79)
		
		SE or 95% CI reported	No	12	(44)	11	(22)	23	(30)
			Yes	15	(56)	38	(78)	53	(70)
	
	Interaction	Results of interaction reported	No	7	(26)	16	(33)	23	(30)
			Yes	20	(74)	33	(67)	53	(70)
		
		If yes, level of detail reported:	No interaction found/p-value only	17	(85)	22	(67)	39	(74)

			Interaction estimate & SE/CI/p-value	3	(15)	11	(33)	14	(26)
Discussion	Interaction	If interaction reported, was it discussed	No	10	(50)	11	(33)	21	(40)
			Yes	10	(50)	22	(67)	32	(60)
	
	Overall interpretation	Appropriate based on data presented	No	4	(15)	0	(0)	4	(5)
			Yes	23	(85)	49	(100)	72	(95)

### Design of included trials

The majority (67/76, 88%) of trials employed a simple 2 × 2 factorial design, and randomised individuals only (48/76, 63%) rather than clusters or a mix of individuals and clusters. Reporting of alllocation was poor, with only just over half (40/76, 53%) giving an adequate description of the randomisation process, and only 31/76 (41%) clearly describing concealment of allocation. Of the 53/76 (70%) trials that provided a sample size calculation, the majority (38/53, 71%) based the estimate on analysis "at the margins", which assumes that there is no interaction between the interventions.

### Analysis of included trials

There was some evidence that reporting of approach to handling attrition and other breaches of protocol was improved in trials published after 2003, with 38/49 (78%) compared 14/27 (52%) applying the intention-to-treat (ITT) principle (risk difference = 26%, 95% CI 4% to 48%, p = 0.021). Most trials (61/76, 80%) correctly adjusted for design variables and baseline values of the outcome in the primary analyses, and in trials where clusters were the unit of allocation, applied appropriate methods of analysis to allow for this (22/28, 79%).

Nearly one third of trials (23/76, 31%) provided neither a CONSORT diagram of participant flow nor adequately described this in the text. In terms of assessing comparability of trial arms at baseline, 12/76 (16%) trials provided either no baseline data or presented it for the study cohort as a whole. Of the 64/76 (84%) that did, the majority provided baseline data "inside the table" (46/64, 72%), with five trials (8%) providing it both "inside the table" and "at the margins".

Most trials (67/76, 88%) provided appropriate descriptive data for the primary outcome(s) at follow up, with similar proportions of trials reporting these data "at the margins" (21/67, 31%), "inside the table" (26/67, 39%) or both (20/67, 30%). There was evidence that trials published after 2003 were more likely to provide appropriate between-group estimates of effect such as differences in means or odds ratios. Forty-three of 49 (88%) trials published after 2003 did this compared with 17/27 (63%) of earlier studies (risk difference = 25%, 95% CI 4% to 45%, p = 0.011). Reporting of precision around such estimates with either standard errors or 95% confidence intervals was also better in later trials (38/49 (78%) versus 15/27 (56%) (risk difference = 22%, 95% CI 0% to 44%, p = 0.046).

Fifty-three trials (70%) reported the results of interaction analyses, although only a minority (14/53, 26%) providing interaction estimates and precision. The remaining 39 trials (74%) provided either only a p-value from the interaction analysis or a statement that no interaction was found. All 14 (100%) trials providing detailed results of the interaction analysis also discussed the interaction in the discussion section of the paper, whereas only 18/39 (46%) providing limited results of the interaction analysis did so. Overall interpretation of the study based on the data presented was appropriate in nearly all trials (72/76, 95%).

## Discussion

We found that reporting of design, analysis and presentation issues that are specific to factorial trials was variable, but there was no evidence that reporting of these (factorial design-specific) aspects was improved for trials published after 2003. Reporting of other important aspects that apply generally to all trials was sometimes inadequate, such as the process of randomisation, including concealment, and participant flow. However, our data suggest that reporting of certain other aspects was improved in later trials, such as using an ITT approach, and providing between-group estimates of effect along with standard errors or confidence intervals.

A strength of our review is the rigorous searching of major electronic databases to identify relevant studies. The absence of MeSH terms to reliably identify trials with factorial designs means that we may have missed some trials that did not contain our search terms in the text. Furthermore, although around 12% of the studies included in our review have a design other than 2 × 2, we cannot be certain whether this is a true reflection of the range of factorial studies undertaken to date or an artefact of our search strategy. The proportion is similar to a previous review that used the same textwords to identify factorial studies [1]. It is interesting that cluster trials do not have a MeSH term either but that crossover trials do. It is also possible that trials conceived and conducted as a factorial design, but with results of each intervention reported in separate publications, would be missed.

Our focus on trials of complex interventions in community settings was partly pragmatic in order to maintain a manageable scope for the review, but was also chosen to reflect the organisation of health care and the type of research that is funded, especially in the UK. The majority of patient contact with health care professionals in the UK National Health Service occurs in community settings [6]. Our review is likely to be relevant to a large section of research effort in the UK and other countries. However we recognise that factorial trials may also be used to evaluate complex interventions in secondary or tertiary care settings, or to evaluate simple interventions in any settings. The quality of reporting of such trials may be different to those included here, and therefore we advise caution if attempting to generalise our results to all factorial trials. Although in most cases it was clear whether a trial should be included in the review, we recognise that we have applied our own definitions of complex interventions and community settings, and that other investigators may have selected a different sample of trials for analysis. Finally, our review covers the period 2000-2009, and trials published since then may affect the results.

Our study adds to the limited existing literature on reporting of factorial trials. McAlister et al's 2003 review focussed only on trials that reported clinically important binary outcomes [1]. The included trials were divided into those of ischaemic heart disease and others, many trials investigated drug treatments only, and were conducted in secondary care settings with clinical patient groups. The latest version of the CONSORT statement [7] recognises that a substantial minority of trials use designs other than two-arm parallel, and that while most of the statement applies equally to all designs, there are additional considerations for each design. The are extensions to CONSORT planned to cover all other designs.

## Conclusions

Reports of factorial trials of complex interventions in community settings vary in the amount of information they provide regarding important methodological aspects of design and analysis. This variability supports the extension of CONSORT guidelines to include the reporting of factorial trials.

## List of abbreviations

CONSORT: Consolidated Standards of Reporting Trials; ITT: Intention-to-treat; MeSH: Medical Subject Heading; MRC: Medical Research Council; RCT: Randomised Controlled Trial.

## Competing interests

The authors declare that they have no competing interests.

## Authors' contributions

AAM and TJP conceived the study. AAM conducted the searches and screened article titles and abstracts to determine retrieval of full papers. MPA conducted the data abstraction, which were then checked by AAM. AAM analysed the data. AAM and MPA wrote the initial draft of the manuscript, with critical input from TJP to later drafts. All authors approved the final version of manuscript.

## Supplementary Material

Additional file 1 **Search strategy (MS Word file)**. Click here for file

Additional file 2 **Characteristics of included studies (MS Excel file)**. Click here for file
