# Supplementary material for: Reporting of factorial trials of complex interventions in community settings: a systematic review
Source: Trials. 2011 Jul 19;12:179. doi: 10.1186/1745-6215-12-179 (PMC3157424; doi:10.1186/1745-6215-12-179)
Supplement: Additional file 1 — Search strategy (MS Word file). [file 1745-6215-12-179-S1.DOC]

MEDLINE

1. randomized-controlled-trial.pt.

2. controlled clinical trial.pt.

3. clinical trial.pt.

4. randomized controlled trial.sh.

5. random allocation.sh.

6. double blind method.sh.

7. single blind method.sh.

8. exp clinical trial/

9. (clin$ adj25 trial$).ti,ab.

10. ((singl$ or doubl$ or tripl$ or trebl$) adj25 (blind$ or mask$ or dummy$)).mp.

11. random$.ti,ab.

12. research design.sh.

13. comparative study.sh.

14. exp evaluation studies/

15. follow up studies.sh.

16. prospective studies.sh.

17. (control$ or prospectiv$ or volunteer$).ti,ab.

18. placebos.sh.

19. placebo$.ti,ab.

20. or/1-19

21. (animals not (human and animals)).sh.

22. 20 not 21

23. factorial.tw.

24. 2 by 2.tw.

25. 2x2.tw.

26. or/23-25

27. factor analysis.tw.

28. 26 not 27

29. 22 and 28

30. remove duplicates from 29

Embase

1. clinical trial.de.

2. controlled clinical trial.de.

3. randomized controlled trial.de.

4. major clinical study.de.

5. double blind procedure.de.

6. single blind procedure.de.

7. randomization.de.

8. placebo.de.

9. prospective study.de.

10. comparative study.de.

11. follow up.de.

12. ((singl$ or doubl$ or tripl$ or trebl$) adj (blind$ or mask$ or dummy$)).ti,ab.

13. randomi#ed.ti,ab.

14. randomly.ti,ab.

15. placebo$.tw.

16. (clinic$ adj (trial$ or study or studies$)).ti,ab.

17. comparative stud$.ti,ab.

18. (control$ or prospectiv$ or volunteer$).ti,ab.

19. or/1-18

20. ((animal or nonhuman) not (human and (animal or nonhuman))).de.

21. 19 not 20

22. factorial.tw.

23. factorial design/

24. 2 by 2.tw.

25. 2x2.tw.

26. or/22-25

27. factor analysis/

28. 26 not 27

29. 21 and 28

PsycINFO

1. exp Treatment Effectiveness Evaluation/

2. clinical trials.de.

3. placebo.de.

4. treatment outcomes.de.

5. evaluation.de.

6. followup studies.de.

7. random$.ti,ab.

8. placebo$.tw.

9. comparative stud$.ti,ab.

10. randomi#ed controlled trial$.ti,ab.

11. (clinical adj3 trial$).ti,ab.

12. (research adj3 design).ti,ab.

13. (evaluat$ adj3 stud$).ti,ab.

14. (prospectiv$ adj3 stud$).ti,ab.

15. ((singl$ or doubl$ or tripl$ or trebl$) adj (blind$ or mask$ or dummy$)).ti,ab.

16. or/1-15

17. animal.po.

18. (human or inpatient or outpatient).po.

19. 17 and 18

20. 17 not 19

21. 16 not 20

22. factorial.tw.

23. 2 by 2.tw.

24. 2x2.tw.

25. or/22-24

26. exp Factor Analysis/

27. 25 not 26

28. 21 and 27
